# The leading methods of suicide in Taiwan, 2002-2008

**DOI:** 10.1186/1471-2458-10-480

**Published:** 2010-08-13

**Authors:** Jin-Jia Lin, Shu-Sen Chang, Tsung-Hsueh Lu

**Affiliations:** 1 Department of Psychiatry, Chi-Mei Medical Center, Tainan, Taiwan; 2 Department of Psychiatry, School of Medicine, College of Medicine, Taipei Medical University, Taipei, Taiwan; 3 Institute of Public Health, College of Medicine, National Cheng Kung University, Tainan, Taiwan; 4 Department of Social Medicine, University of Bristol, Bristol, UK; 5 Department of psychiatry, Ju Shan Hospital, Taoyuan, Taiwan

## Abstract

**Background:**

Diverse socioeconomic and cultural developments between geographic regions and cities/counties have resulted in different physical availability and socio-cultural acceptability of certain methods of suicide. This study examined the changes in distribution of the leading methods of suicide across cities/counties in Taiwan between 2002-04 and 2006-08.

**Methods:**

Mortality data for all deaths classified as suicide or as of undetermined intent from 2002 through 2008 were extracted for analysis. The number of deaths and proportion of completed suicides by four main methods were calculated in order to identify the leading lethal methods in each city/county.

**Results:**

Hanging was the leading method of suicide in 18 out of 22 cities/counties in 2002-04 but decreased to 10 out of 22 in 2006-08. On the other hand, charcoal burning was not the leading method in any city/county in 2002-04 but increased to 10 out of 22 in 2006-08. The younger the age of the deceased, the more likely the leading method of suicide changed from 2002-04 to 2006-08. Charcoal burning was the most often used method in most cities/counties among those aged 15-44; however, hanging was most frequent for those aged 45 or above. Pesticides were the leading method for the elderly in five counties with a high percentage of agricultural population in 2006-08.

**Conclusion:**

The leading method of suicide varied by age group and changed from 2002-04 to 2006-08 in Taiwan. This was due primarily to changes in socio-cultural acceptability of the use of charcoal burning as a method for suicide by younger age groups.

## Background

Restricting the methods of suicide has been proposed as one of the most effective suicide prevention strategies [1-5]. As physical availability and socio-cultural acceptability are two important determinants in the choice of method [6], these factors would differ across geographic areas and therefore the leading methods of suicide would differ accordingly. Ajdacic-Gross *et al. *used mortality data from the World Health Organization to illustrate great variations in the preferred method of suicide across countries [7]. For example, poisoning by pesticides was common in some Asian countries (e.g., 38% of all suicides in Korea) and in Latin America (e.g., 86% in El Salvador, 61% in Nicaragua and 55% in Peru); poisoning by drugs was common in both Nordic countries (e.g., 18% in Finland and 14% in Demark) and the United Kingdom (15%); hanging was the preferred method of suicide in Eastern Europe (e.g., 92% in Lithuania, 85% in Latvia and 70% in Slovakia); as was suicide with firearms in the United States (61%) and jumping from a high place in cities and urban societies such as Hong Kong (43%) [7].

Ajdacic-Gross *et al. *further commented that the patterns of suicide typically change very slowly except for suicide by charcoal burning [7]. Suicide by charcoal burning has become epidemic in Hong Kong, Taiwan and Japan [8-12]. In Taiwan, only 32 people completed suicide by charcoal burning in 1998 but this number increased to 1346 in 2005 [10]. A recent spatial and temporal analysis suggested that the epidemic of suicide by charcoal burning in Taiwan emerged more prominently in urban areas, was without a single point of origin, and rates remained highest in metropolitan regions over time [11].

Little is known about the differences in suicide rates by charcoal burning by geographic regions and cities/counties. This would have different implications for suicide prevention programs. For example, Taipei City and Kaohsiung City are the only two metropolitan areas in Taiwan and are classified at the same level of urbanization; however, Taipei City is in northern Taiwan and Kaohsiung City in southern Taiwan and the city governments were run by different political parties with many different local policies. City/county is the most important political administrative unit in Taiwan with its own budget and autonomy in designing locally relevant suicide prevention programs.

Cities/counties in Taiwan differ greatly in socio-demographic characteristics, ethnic composition, dialects used, economic structure and income level (Table 1). Taiwan experienced rapid economic growth during the 1970s and 1980s and this resulted in diverse development between cities/counties. For example, many high technology industries are located in northern Taiwan (such as Taipei County, Tauyuan County and Hsinchu County); and many counties (e.g., Nantou County, Yunlin County, Chiayi County, Pintung County, and Taitung County) rely predominantly on traditional industries and have a high percentage of agricultural population and lower disposable household income (Table 1). All seven cities are in urban areas with high population density. Most counties include some urban townships with high population density and some rural townships with low population density. General speaking, counties in the south and east have more rural townships and are poorer.

**Table 1:** Socioeconomic characteristics* and suicide rates for each city/county in Taiwan, 2005

Geographic region City/county	Population	**Population density, pop/km**^**2**^	Primary industry, %	Secondary industry, %	Tertiary industry, %	Disposable household income, USD	Suicide rate, per 100,000
**North**							
Taipei City	2,616,375	9626	0.2	19.3	80.5	37455	17.7
Keelung City	391.727	2951	0.6	28.1	71.3	26520	32.8
Hsinchu City	390,692	3753	1.2	42.5	56.3	33104	23.5
Taipei County	3,736,677	1820	0.7	37.8	61.5	28309	25.0
Taoyuan County	1,880,316	1540	2.0	46.2	51.8	29865	24.6
Hsinchu County	477,677	335	3.6	51.9	44.5	31915	20.2
Yilan County	461,586	215	7.3	34.1	58.6	24924	23.7
**Middle**							
Taichung City	1,032,778	6320	0.6	28.0	71.4	26680	22.8
Miaoli County	559,944	308	7.5	46.7	45.8	23299	23.8
Taichung County	1,533,442	747	5.3	47.8	46.9	23892	24.2
Changhua County	1,315,826	1225	10.8	44.8	44.4	23195	22.2
Nantou County	537,168	131	18.0	30.1	51.9	22681	31.3
Yunlin County	733,330	568	23.5	30.2	46.3	19657	26.2
**South**							
Kaohsiung City	1,510,649	9835	0.8	31.9	67.3	28956	24.9
Tainan City	756,859	4309	1.5	37.3	61.2	24593	21.7
Chiayi City	271,701	4526	2.1	25.5	72.5	24633	16.8
Chiayi County	557,101	293	25.7	31.3	43.0	20578	28.2
Tainan County	1,106,059	549	12.2	42.9	44.9	20698	23.6
Kaohsiung County	1,242,837	445	8.2	40.0	51.8	22382	24.6
Pingtung County	898,300	324	18.4	26.3	55.3	22457	27.9
**East**							
Hualien County	347,298	75	12.0	24.5	63.5	20083	29.8
Taitung County	238,943	68	22.4	24.8	52.9	18104	21.6

* access from

The diverse economic and social development between geographic regions and cities/counties has resulted in diverse physical availability and socio-cultural acceptability of certain methods of suicide. A previous study in Taiwan indicated that cities/counties with a higher percentage of agricultural population were more likely to have suicides from pesticides and cities/counties with a higher percentage of high buildings were more likely to have suicides from jumping [13]. We hypothesize that the leading method of suicide would be different across cities/counties and would have changed after the epidemic of suicide by charcoal burning.

## Methods

### Mortality data

This study used officially published data, therefore no approval is required. All deaths classified as suicide or of undetermined intent from 2002 to 2008 were extracted from the database of the Department of Health of the Executive Yuan of Taiwan for analysis. We confined this study to the years 2002-2008 because the International Classification of Diseases, Tenth Revision (ICD-10) was used beginning in 2002 and this provides a better classification of methods of suicide (especially pesticide poisoning) in the mortality data. The national coders in Taiwan used only 3-digit ICD-9 codes in tabulating national cause-of-death statistics before 2002. We could not differentiate suicide by pesticide poisoning (ICD-9 code E950.6) from suicide by solid or liquid poisoning (ICD-9 code E950) in Taiwan before 2002.

### Analysis

According to a previous Taiwanese study [9], the four most commonly employed methods of suicide were hanging (ICD-10 code X70 and Y20), charcoal burning (ICD-10 code X67 and Y67), pesticide poisoning (ICD-10 code X68 and Y18), and jumping from heights (ICD-10 code X80 and Y30). Deaths among those aged 14 and below were not included in the analysis. To better understand the distribution of various types of pesticides used in Taiwan, we requested Office of Statistics, Department of Health to provide statistics of types of the pesticides reported on death certificate for years 2006-08.

The unit of analysis in this study is the city/county. There are 7 cities and 18 counties in Taiwan and cities usually have a higher level of urbanization and population density than counties (Table 1). Three counties, i.e., Penghu County, Kinmen County, and Lienchiang County, were excluded from this analysis because of their small populations.

To increase the stability of the number of deaths for cities or counties with relatively small populations, we combined three years of data for analysis as we had only 7 years of data and the largest time window to examine the changes would be from 2002-04 to 2006-08. We first computed the number of deaths and the proportion of completed suicides by the four most commonly used methods for each city/county in 2002-2004 and 2006-2008. We then calculated the proportion of suicides committed by the leading methods among all suicides and deaths of undetermined intent in each city/county by sex and age (15-24, 25-44, 45-64, and 65 and older). We used ArcGIS Version 9.3 to illustrate graphically the regional variations in the leading methods of suicide across years and by age group.

## Results

**Figure 1:**
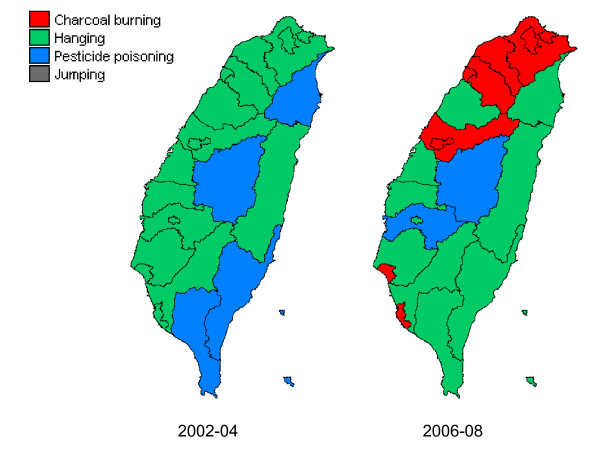
**Leading method of suicide in each city/county in Taiwan, 2002-04 and 2006-08**.

For Taiwan as a whole, hanging was the leading method of suicide in both 2002-04 and 2006-08 and accounted for 30% of all suicides and deaths of undetermined intent. At the city/county level, hanging was the leading method in 18 out of 22 cities/counties in 2002-04 but decreased to 10 out of 22 cities/counties in 2006-08 (Table 2 and Figure 1). On the other hand, charcoal burning was not the leading method in any city/county in 2002-04 but increased to 10 out of 22 in 2006-08. Charcoal burning became the most commonly used method of suicide in 6 cities and 4 prosperous counties (i.e., Taipei County, Taoyuan County, Hsinchu County, and Taichung County) in 2006-08.

**Table 2:** Number of deaths (No) and percentage (%) of three most commonly used methods of suicide in each city/county in Taiwan, 2002-04 and 2006-08 (the leading method of suicide in each city/county is highlighted)

		Hanging		Charcoal burning		Pesticides		All suicide	
					
City/county	Year	No	%	No	%	No	%	No	%
Taiwan	2002-04	**3800**	**31**	2309	19	1785	15	12173	100
	2006-08	**4280**	**30**	4110	29	1651	12	14248	100
**North**									
Taipei City	2002-04	**358**	**31**	240	21	39	3	1169	100
	2006-08	388	30	**394**	**30**	23	2	1304	100
Keelung City	2002-04	**97**	**31**	68	22	24	8	316	100
	2006-08	107	30	**121**	**33**	13	4	362	100
Hsinchu City	2002-04	**66**	**36**	45	25	17	9	183	100
	2006-08	57	28	**75**	**36**	10	5	206	100
Taipei County	2002-04	**573**	**31**	478	26	115	6	1865	100
	2006-08	731	31	**794**	**33**	82	3	2391	100
Taoyuan County	2002-04	**303**	**31**	233	23	151	15	993	100
	2006-08	303	27	**367**	**33**	118	11	1104	100
Hsinchu County	2002-04	**62**	**30**	33	16	54	26	205	100
	2006-08	82	27	**96**	**31**	69	22	307	100
Yilan County	2002-04	90	30	35	12	**97**	**33**	297	100
	2006-08	**99**	**31**	74	23	80	25	323	100
**Middle**									
Taichung City	2002-04	**146**	**28**	141	27	37	7	514	100
	2006-08	163	28	**214**	**36**	21	4	591	100
Miaoli County	2002-04	**95**	**30**	43	14	87	27	318	100
	2006-08	**115**	**31**	84	23	97	26	371	100
Taichung County	2002-04	**238**	**30**	166	21	138	17	802	100
	2006-08	278	28	**302**	**31**	132	13	983	100
Nantou County	2002-04	101	29	41	12	**141**	**40**	353	100
	2006-08	114	27	104	25	**126**	**30**	419	100
Changhua County	2002-04	**191**	**30**	113	18	102	16	628	100
	2006-08	**221**	**30**	174	23	146	20	742	100
Yunlin County	2002-04	**142**	**32**	58	13	140	32	444	100
	2006-08	**150**	**29**	112	22	132	26	509	100
**South**									
Kaohsiung City	2002-04	**256**	**29**	176	20	22	3	868	100
	2006-08	277	28	**323**	**32**	26	3	998	100
Tainan City	2002-04	**154**	**41**	66	17	18	5	380	100
	2006-08	147	33	**159**	**35**	11	2	450	100
Chiayi City	2002-04	**51**	**37**	28	20	12	9	139	100
	2006-08	**49**	**32**	35	23	6	4	154	100
Chiayi County	2002-04	**100**	**32**	41	13	89	28	314	100
	2006-08	106	27	67	17	**115**	**30**	389	100
Tainan County	2002-04	**245**	**37**	85	13	128	20	654	100
	2006-08	**245**	**34**	178	25	103	14	712	100
Kaohsiung County	2002-04	**228**	**32**	113	16	90	13	710	100
	2006-08	**276**	**34**	219	27	92	11	819	100
Pingtung County	2002-04	140	27	49	9	**154**	**30**	520	100
	2006-08	**179**	**30**	123	21	139	23	592	100
**East**									
Hualien County	2002-04	**94**	**34**	29	11	76	28	276	100
	2006-08	**98**	**33**	62	21	68	23	293	100
Taitung County	2002-04	41	24	19	11	**52**	**31**	168	100
	2006-08	**60**	**38**	22	14	40	25	157	100

Table 3 shows the leading methods of suicide by city/county, sex and age between 2002-04 and 2006-08 in Taiwan. The sex-age groups whose leading method changed from 2002-04 to 2007-08 are highlighted. We found that the younger the age of the deceased, the more likely that the leading method would change from 2002-04 to 2006-08. Of 44 sex-city/county groups, 28 showed changes in the leading method for those aged 15-24. For the 25-44 age group, 18 showed changes as did 16 for the 45-64 group, and 13 for the ≧65 group. For females aged 15-24, the leading method of suicide was jumping in 9 cities/counties in 2002-04 but remained so in only one city (i.e., Taipei city) in 2006-08 as suicidal individuals in most cities/counties changed to charcoal burning.

**Table 3:** The leading method of suicide (C = charcoal burning, H = hanging, J = Jumping, P = pesticides) and percentage of all suicides (%) by city/county, sex, age and year in Taiwan (the sex-age groups where the leading method of suicide changed from 2002-04 to 2006-08 are highlighted)

		Total		15-24 yrs		25-44 yrs		45-64 yrs		≧65 yrs	
						
City/county	Sex	02-04	06-08	02-04	06-08	02-04	06-08	02-04	06-08	02-04	06-08
Taiwan	M	H (33)	H (32)	**H (31)**	**C (39)**	C (31)	C (45)	H (34)	H (34)	H (47)	H (45)
	F	H (27)	H (27)	**J (31)**	**C (34)**	C (30)	C (40)	H (30)	H (29)	H (37)	H (38)
**North**											
Taipei City	M	**H (32)**	**C (34)**	**H (38)**	**C (46)**	C (34)	C (47)	**H (30)**	**C (33)**	H (50)	H (48)
	F	**J (29)**	**H (28)**	J (44)	J (40)	**J (31)**	**C (37)**	H (32)	H (29)	H (40)	H (40)
Keelung City	M	H (32)	H (33)	**J (43)**	**H (29)**	C (38)	C (47)	H (27)	H (37)	H (47)	H (43)
	F	**H (28)**	**C (36)**	**J (33)**	**C (60)**	**J (29)**	**C (52)**	**H (32)**	**C (37)**	H (38)	H (44)
Hsinchu City	M	**H (38)**	**C (38)**	H (46)	H (73)	C (44)	C (49)	**H (54)**	**C (38)**	H (39)	H (48)
	F	**H (31)**	**C (34)**	**J (33)**	**C (75)**	C (58)	C (44)	**H (20)**	**C (30)**	H (73)	H (31)
Taipei County	M	**H (31)**	**C (37)**	C (27)	C (41)	C (37)	C (49)	H (33)	H (34)	H (55)	H (53)
	F	H (29)	H (28)	**J (36)**	**C (35)**	C (34)	C (33)	H (38)	H (29)	H (47)	H (54)
Taoyuan County	M	**H (31)**	**C (34)**	**H (33)**	**C (33)**	C (36)	C (49)	H (27)	H (31)	H (51)	H (40)
	F	**H (29)**	**C (32)**	C (30)	C (43)	C (37)	C (49)	**H (35)**	**C (27)**	**P (30)**	**H (33)**
Hsinchu County	M	**H (33)**	**C (33)**	C (36)	C (54)	**H (28)**	**C (46)**	**P (37)**	**H (30)**	H (52)	H (36)
	F	**P (29)**	**H (30)**	**C (20)**	**H (40)**	C (33)	C (36)	**P (36)**	**C (37)**	P (36)	P (39)
Yilan County	M	**P (35)**	**H (29)**	**P (33)**	**C (58)**	**H (36)**	**C (36)**	**P (43)**	**H (31)**	**P (40)**	**H (42)**
	F	H (30)	H (33)	**C (57)**	**H (22)**	**P (32)**	**C (36)**	H (36)	H (33)	H (37)	H (52)
**Middle**											
Taichung City	M	**H (32)**	**C (40)**	C (32)	C (43)	C (39)	C (52)	H (36)	H (38)	H (47)	H (44)
	F	C (30)	C (28)	**J (33)**	**C (25)**	C (43)	C (42)	H (34)	H (23)	H (35)	H (30)
Miaoli County	M	H (30)	H (31)	H (50)	H (35)	H (26)	H (31)	H (31)	H (29)	P (34)	P (46)
	F	H (30)	H (31)	H (33)	H (27)	**H (30)**	**C (36)**	**P (44)**	**H (42)**	**H (30)**	**P (49)**
Taichung County	M	**H (30)**	**C (32)**	C (30)	C (47)	C (32)	C (48)	H (30)	H (35)	H (48)	H (46)
	F	**H (29)**	**C (28)**	**J (33)**	**C (45)**	C (29)	C (44)	H (33)	H (34)	H (40)	H (25)
Nantou County	M	P (37)	P (31)	**H (47)**	**C (31)**	**H (33)**	**C (42)**	P (45)	P (35)	**H (42)**	**P (42)**
	F	P (46)	P (27)	**H (29)**	**J (50)**	**P (39)**	**C (40)**	**P (56)**	**H (27)**	P (48)	P (43)
Changhua County	M	H (33)	H (32)	H (41)	H (36)	C (34)	C (37)	H (36)	H (32)	**H (40)**	**P (39)**
	F	H (24)	H (25)	C (50)	C (31)	C (26)	C (43)	**P (29)**	**H (30)**	**H (32)**	**P (26)**
Yunlin County	M	H (32)	H (28)	**H (36)**	**C (73)**	**P (29)**	**C (40)**	H (37)	H (32)	**H (38)**	**P (41)**
	F	**P (32)**	**H (34)**	**J (44)**	**C (43)**	**H (28)**	**C (40)**	H (42)	H (34)	**P (43)**	**H (48)**
**South**											
Kaohsiung City	M	**H (34)**	**C (33)**	**C (45)**	**J (35)**	C (32)	C (50)	H (41)	H (37)	H (43)	H (44)
	F	**J (26)**	**C (31)**	**J (35)**	**C (55)**	C (32)	C (45)	H (26)	H (28)	**J (27)**	**H (30)**
Tainan City	M	H (41)	H (36)	H (32)	H (43)	C (33)	C (55)	H (48)	H (42)	H (72)	H (56)
	F	**H (40)**	**C (34)**	**H (40)**	**C (50)**	C (25)	C (54)	**H (48)**	**C (27)**	H (53)	H (50)
Chiayi City	M	H (36)	H (34)	**H (80)**	**C (67)**	C (34)	C (37)	H (35)	H (41)	H (39)	H (41)
	F	H (38)	H (27)	**C (50)**	**H (50)**	C (33)	C (38)	H (50)	H (39)	H (59)	H (17)
Chiayi County	M	H (33)	H (29)	**H (46)**	**C (27)**	C (29)	C (34)	H (30)	H (32)	**H (43)**	**P (45)**
	F	**H (29)**	**P (31)**	H (29)	H (25)	**P (24)**	**C (29)**	P (41)	P (26)	**H (50)**	**P (42)**
Tainan County	M	H (40)	H (38)	**H (38)**	**C (48)**	**H (28)**	**C (43)**	H (45)	H (42)	H (51)	H (53)
	F	H (31)	H (26)	**H (45)**	**J (41)**	C (23)	C (40)	H (23)	H (23)	H (42)	H (39)
Kaohsiung County	M	H (35)	H (34)	**H (42)**	**C (37)**	**H (27)**	**C (43)**	H (37)	H (39)	H (46)	H (48)
	F	H (25)	H (33)	H (33)	H (38)	C (31)	C (33)	H (27)	H (36)	H (34)	H (41)
Pingtung County	M	H (31)	H (32)	H (43)	H (33)	**P (31)**	**C (37)**	**P (39)**	**H (30)**	H (43)	H (37)
	F	**P (27)**	**H (27)**	H (42)	H (33)	**H (24)**	**C (44)**	**P (35)**	**H (31)**	**P (31)**	**H (30)**
**East**											
Hualien County	M	H (39)	H (35)	**C (27)**	**J (43)**	**H (35)**	**C (30)**	H (44)	H (36)	H (46)	H (54)
	F	P (32)	P (30)	**P (40)**	**H (50)**	**P (30)**	**C (29)**	**P (32)**	**H (29)**	**H (40)**	**P (52)**
Taitung County	M	**P (31)**	**H (39)**	**P (50)**	**C (40)**	**P (31)**	**H (40)**	P (39)	P (43)	H (46)	H (51)
	F	**P (30)**	**H (37)**	**J (40)**	**H (33)**	C (31)	C (38)	**P (45)**	**H (58)**	P (40)	P (54)

**Figure 2:**
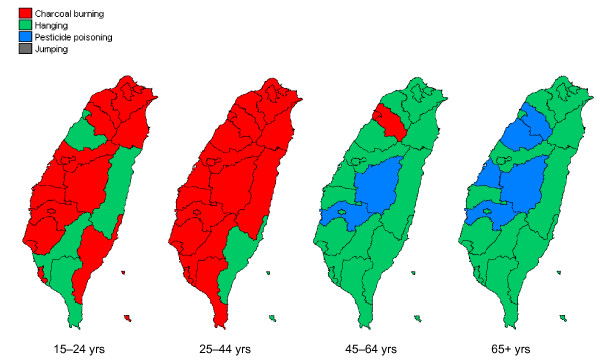
**Leading method of suicide in each city/county by age group in Taiwan, 2006-08**.

The distribution of leading methods of suicide across cities/counties differed greatly by age group (Figure 2). In 2006-08, for 17 out of 22 cities/counties, charcoal burning was the leading method for those aged 15-24 and this number increased to 21 out of 22 cities/counties for 25-44 year olds. Hanging was still the most commonly used method for 45-64 year olds (18 out of 22) and ≧65 years olds (17 out of 22). Of elderly females who completed suicide in eastern Taiwan (i.e., Hualien County and Taitung County) more than half used pesticides to kill themselves.

Of 1651 suicides by pesticides occurred between 2006 and 2008, 541 (32.8%) cases used herbicides, 306 (18.5%) cases used insecticides, 38 (2.3%) cases used other pesticides, and 766 (46.4%) cases did not report specified type of pesticides. Parauqat was the most common used herbicides (471 cases) and organophosphates (130 cases) and methomyl (97 cases) were the two most often used insecticides in Taiwan.

## Discussion

### Main findings

Our findings suggest large changes in the distribution of leading methods of suicide across cities/counties in Taiwan from 2002-04 to 2006-08, the period in which charcoal burning became epidemic. The distribution differed greatly by age group. For those aged 15-44, charcoal burning was the leading method of suicide in most cities/counties. For those aged 45 or above, hanging was the leading method in most cities/counties. Pesticides were the leading method for the elderly in some cities/counties with a higher percentage of agricultural population.

### Physical availability vs. socio-cultural acceptability

Cantor and Baume suggested that physical availability and socio-cultural acceptability are two important determinants of the choice of a method of suicide [6]. However, few studies have assessed the relative influence of these two determinants in choosing a particular method. An examination of geographic variations and changes over time of the leading method of suicide could provide some hints as to the relative importance of the two determinants.

According to the classification proposed by Marzuk *et al.*[14], firearms, high buildings and pesticides are differentially accessed methods of suicide and choice is influenced by physical availability. The study by Ajdacic-Gross *et al. *showed that the leading method was firearms in the United States, jumping from high places in Hong Kong and pesticides in some Asian countries and in Latin America. These are examples of differential physical availability [7]. Other examples are that cities/counties in Taiwan with a higher percentage of agricultural population (a proxy for the availability of pesticides) were more likely to have higher suicide rates from pesticides and cities/counties with a higher percentage of high buildings were more likely to have higher suicide rates from jumping [13].

Those two studies, however, did not determine whether there were differences in leading methods of suicide by age group. The findings of this study suggest that within the counties with a high percentage of agricultural population (i.e., equal physical availability of pesticides to all age groups), we still found large differences in the use of pesticides as the method of suicide by different age groups. Only the elderly were more likely to choose pesticides in these counties; this might be due to differences in socio-cultural acceptability of certain methods of suicide in particular age groups.

This study also found age group differences and changes over time in the socio-cultural acceptability of charcoal burning. Charcoal burning (an equally physically available method of suicide for different age groups over the years) is more acceptable to people aged 15-44 than to their counterparts aged 45 and above. In 2002-04, charcoal burning was not the leading method of suicide in any city/county but it increased to 10 out of 22 in 2006-08. Of the 9 cities/counties in which jumping was the leading method among females aged 15-24 in 2002-04, 8 changed to charcoal burning in 2006-08. These findings suggest an increasing socio-cultural acceptance of the use of charcoal burning as a method of suicide by younger people over the years.

Other evidence supporting the importance of socio-cultural acceptability was the huge difference in the percentage of persons using hanging as the method of suicide across countries. This ranged from 8.4% in El Salvador to 91.7% in Kuwait [7]. This study further revealed that the percentage of those using hanging varied across cities/counties and by age group. As rope is a universally-available tool for hanging in every country and for all age groups, the difference in the percentage of people choosing hanging were due mainly to differences in its socio-cultural acceptability by geographic region and age group.

### Strengths and limitations

To the best of our knowledge, this is the first study to assess the changes in distribution of the leading methods of suicide by sex-age groups at the city/county level within a single country. The surge in the use of charcoal burning as the method of choice in the past decade in Taiwan provides an opportunity to examine the relative role of socio-cultural acceptability in that choice. The centralized civil and vital registration system in Taiwan allowed us to have standardized and uniform mortality data for regional comparisons.

One of the limitations in examining the leading methods of suicide instead of suicide rates was the reduction of interval scale data into categorical scale data. In many cities and counties there might be only a small differences in the number of deaths between the first and the second most commonly used methods; however, only the first was included for analysis in this study. Despite this limitation, the leading method of suicide, similar to the leading cause of death [15], is a very simple and easily-understood indicator used to express the importance of the problem to the general public and to health policy-makers. In addition, the focus on the leading method made it possible to summarize the vast amount of information garnered across regions, sex-age groups and years.

A second limitation was the possible bias in regional variation in underreporting deaths by suicide. To avoid bias in underreporting across regions, we included deaths of undetermined intent in the analysis. A previous study in Taiwan indicated that relatively few deaths by suicide were reported as being committed by an unspecified method. This did not vary across regions [16].

### Implications for suicide prevention

Restricting the methods for suicide has been proposed as one of the most effective suicide prevention strategies [1-5]. Nevertheless, most discussion has focused on the restriction of physical availability and relatively little attention has been paid to alteration of the socio-cultural acceptability of certain methods. Socio-cultural acceptability, as indicated by Cantor and Baume, is a measure of the extent to which a person's choice of method is shaped and circumscribed by the norms, traditions, and moral attitudes of their culture [6]. The findings of this study suggest that socio-cultural acceptability still plays a very important role in determining the choice of method (e.g., charcoal burning).

Florentine and Crane proposed that the concept of 'cognitive availability', i.e., how accessible something is in one's mind, can also play a role in choosing the method of suicide [5]. They emphasized that the media can increase cognitive availability of a particular method by distributing technical information about how to enact that method, by sensationalizing it, and by giving inaccurate portrayals that may encourage its use. They used charcoal burning as an example to illustrate how 'cognitive availability' might be differently shaped by news reports in Hong Kong. Despite the physical availability of charcoal in western counties, as pointed by Florentine and Crane, there are currently very few suicides by this method in western countries because charcoal is not culturally associated with suicide [5].

A previous study indicated that charcoal burning has been portrayed as an easy, painless and attractive way to die. Furthermore, charcoal burning, when compared with hanging and jumping, is more compatible with the traditional Chinese belief emphasizing preservation of the complete corpse for burial, as this ensures a good beginning for the next incarnation [17]. Further qualitative studies are needed to better understand the different degrees of socio-cultural acceptability in choosing charcoal burning as the method of suicide in different age groups. This information is essential in order to design social marketing strategies to discourage the acceptability of using charcoal burning as method of suicide.

## Conclusions

Large variations in the distribution of leading methods of suicide across geographic regions and cities/counties in Taiwan were partially due to the different physical availability of particular methods such as pesticides and high buildings; however, great differences in patterns between age groups and across time within a given city/county (equal physical availability) were due mainly to differences and changes in the socio-cultural acceptability of certain methods (especially charcoal burning and pesticides). Suicide prevention programs at the city/county level should not only consider restricting the locally relevant, physically available methods of suicide, but should also consider addressing the false socio-cultural acceptability of certain methods of suicide by particular age groups.

## Competing interests

The authors declare that they have no competing interests.

## Authors' contributions

JJL contributed to the study design, analysis and interpretation of the data and drafted the paper. SSC contributed to the analysis and interpretation of the data. THL contributed to the study design, obtained the data and commented on the interpretation. All authors have read and approved the final manuscript.

## Pre-publication history

The pre-publication history for this paper can be accessed here:

http://www.biomedcentral.com/1471-2458/10/480/prepub
